# CCL signaling drives T Cell–Macrophage crosstalk in the mouse colon during chronic *Trypanosoma cruzi* infection

**DOI:** 10.1016/j.isci.2026.116611

**Published:** 2026-07-02

**Authors:** Erica Silberstein, Spyros Karaiskos, Nikki Tirrell, Charles C. Chung, Supriya Kumar, Jung-Sun Cho, Alain Debrabant

**Affiliations:** 1 Laboratory of Emerging Pathogens, Office of Blood Research and Review, Center for Biologics Evaluation and Review, Food and Drug Administration, Silver Spring, MD, USA; 2 High-Performance Virtual Environment (HIVE), Office of Biostatistics and Pharmacovigilance, Center for Biologics and Evaluation, Silver Spring, MD, USA

**Keywords:** chronic digestive Chagas disease, Trypanosoma cruzi, colonic lamina propria cells, scRNA-seq, CD8⁺ cytotoxic T cells, macrophages, immune cell communication, CCL5-CCR5 axis

## Abstract

Chagas disease, caused by *Trypanosoma cruzi*, progresses through acute and chronic phases and is characterized by parasite persistence in multiple tissues, including the heart, gastrointestinal tract, and skeletal muscle. Digestive Chagas disease (DCD) is associated with pathological alterations in the colon during chronic infection. To characterize the immune landscape underlying this process, we used a murine model of chronic DCD and performed single-cell RNA sequencing of colonic lamina propria cells. Our analysis revealed infiltration of T cells, B cells, and macrophages, with T cells representing the predominant immune population in the chronically infected colon. Computational inference of cell-cell communication predicted strong activation of the CCL chemokine signaling pathway, with the CCL5-CCR5 axis emerging as a potential mediator of interactions between CD8^+^ cytotoxic T cells and macrophages. These findings highlight the importance of chemokine-driven immune coordination in chronic infection and provide a foundation for the identification of biomarkers and the development of targeted therapeutic strategies for chronic DCD.

## Highlights

• Chronic *Trypanosoma cruzi* infection drives robust immune cell recruitment to the mouse colon
• T cells are the predominant immune cell population in the chronically infected colon
• The CCL5-CCR5 chemokine axis may mediate immune cell communication
• CD8^+^ cytotoxic T cells - macrophage crosstalk is predicted to shape local immune responses

## Graphical abstract

**Figure:**
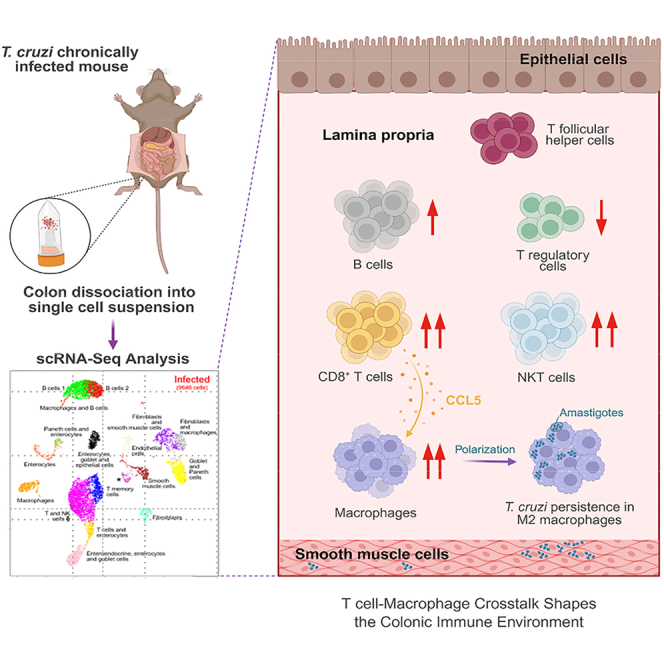

## Teaser

Immunology; cell biology

## Introduction

*Trypanosoma cruzi (T. cruzi)* is the etiological agent of Chagas disease (CD), infecting 6–7 million individuals worldwide.^1^ An estimated 300,000 infected individuals live in the United States, most of whom acquired the infection in endemic regions of Latin America. Local transmission occurs mainly by triatomine bugs and congenitally from mother to child. The disease has an acute phase that lasts 4–8 weeks and is characterized by an effective immune response that reduces the parasite burden.^2^^,^^3^ However, infected hosts fail to completely clear the infection and progress to a chronic phase during which *T. cruzi* persists mainly in the heart, gastrointestinal (GI) tract, and skeletal muscles. During this lifelong chronic stage, while most individuals exhibit no symptoms throughout their lives, about 30% may develop CD cardiomyopathy (CDC) and/or digestive CD (DCD).

The most common GI manifestations of CD are megacolon and megaesophagus syndromes that result from the progressive dilation and dysfunction of these organs, leading to various symptoms such as difficulty swallowing, abdominal pain, and severe constipation. Dilation is linked to the damage of enteric neurons, which causes peristaltic paralysis and smooth muscle hypertrophy, impacting GI tract motility.^4^^,^^5^ More recently, interstitial cells of Cajal -critical for coordinating motility patterns-have also been linked to colon dysfunction in a murine model of chagasic megacolon.^6^

Nevertheless, critical gaps remain in our knowledge of the mechanisms driving DCD. Studies on parasite-host interactions represent an active area of research essential for advancing vaccine development, identifying diagnostic biomarkers, designing effective therapies, and understanding why some people experience severe disease while others remain symptom-free. A balanced immune response is needed to effectively control parasite replication while preventing excessive activation and minimizing tissue damage.^7^

Several murine DCD models are currently available to study host-parasite interactions in the chronically infected GI tract.^8^^,^^9^ Using bioluminescent *T. cruzi* strains combined with *in vivo* and *ex vivo* bioluminescence imaging, we and others have identified the intestine as a major parasite reservoir in chronically infected mice.^10^^,^^11^ Subsequent investigations have shown that most parasites reside in the circular muscle layer of the colon, contributing to tissue pathology by triggering the degeneration of enteric neurons, delayed gut transit, and inflammation.^8^^,^^12^^,^^13^

In most hosts, *T. cruzi* infection is controlled throughout both the acute and chronic stages by a strong and effective immune response. Macrophages, dendritic and natural killer (NK) cells serve as the first line of defense during the acute phase of infection, followed by the expansion of CD8^+^ and CD4^+^ T cells. CD8^+^ T cells reduce parasite loads to nearly undetectable levels while CD4^+^ helper and follicular helper T cells stimulate B lymphocyte proliferation and antibody production.^14^^,^^15^ Cytokines and co-stimulatory molecules modulate the magnitude and functionality of the T cell response.^16^ Despite the activation of these host defense mechanisms, complete parasite clearance is not achieved. Failure to eliminate the parasite results in the long-term persistence of *T. cruzi* within the intestinal tract, which in turn triggers chronic inflammation and neuronal death.^8^^,^^13^^,^^17^

Previous studies have shown that insufficient recruitment of T cells to infection foci in the colon allows limited parasite growth at sufficient levels to sustain a long-term infection.^18^ Yet, the mechanisms associated with the colonic immune response to *T. cruzi* remain to be elucidated.

In this study, we investigated the single-cell transcriptome landscape of the chronically infected mouse colon, focusing on the lamina propria (LP) compartment where both innate and adaptive immune cells reside.^19^ Computational analysis revealed several distinct cell clusters, with lymphocytes accounting for more than half of these populations. Cell-to-cell communication analysis among immune cells predicted the activation of the C–C motif chemokine ligand (CCL) signaling pathway and identified the main sender and receiver populations. Together, our results underscore the critical role of chemokine networks in long-term inflammation during DCD. More importantly, this study is the first to employ single-cell transcriptomics to explore the *T. cruzi*-infected colon, laying the groundwork for future investigations into immunoregulatory molecules and pathways, as well as aiding in the identification of biomarkers for chronic Chagas disease.

## Results

### Immune cells are recruited to the colon of *T. cruzi* chronically infected mice

The GI tract serves as a known reservoir for *T. cruzi* in mice chronically infected with CD.^10^^,^^11^ Specifically, the parasite persists in myocytes within the colonic gut wall, where they drive a robust cellular immune response that is effective in controlling the infection, but insufficient to fully eliminate *T. cruzi* from the host.^18^ To identify the immune cell populations that drive parasite persistence in the mouse colon during chronic *T. cruzi* infection, we infected C57BL/6J mice with TcCOL-NLuc,^10^ a reporter parasite strain that expresses nanoluciferase. This animal model has been previously validated and successfully used to study DCD.^9^

**Figure 1:**
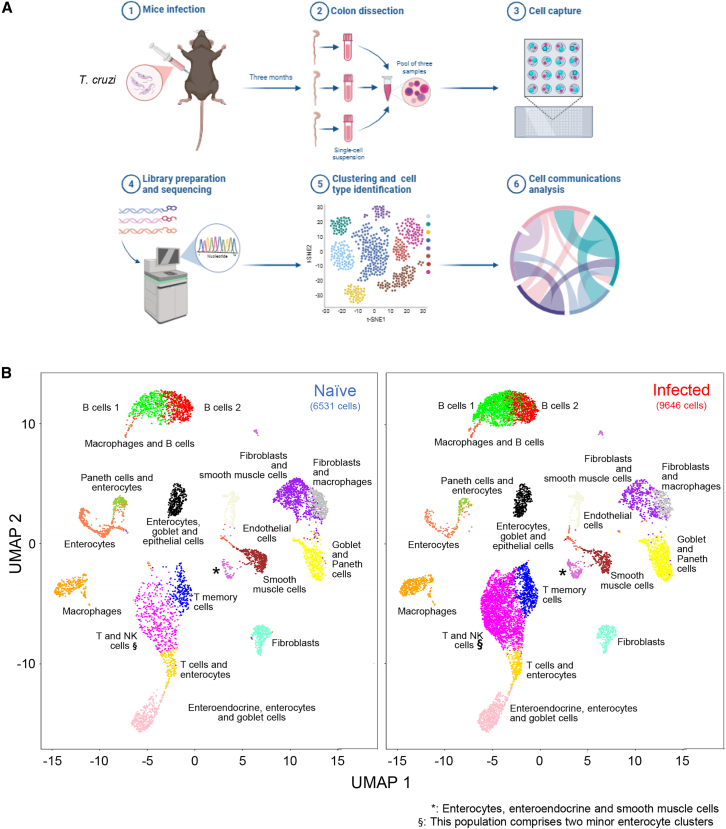
Immune cells are recruited to the colon of *T. cruzi* chronically infected mice (A) Experimental workflow for single-cell RNA sequencing analysis. ① C57BL/6 mice were infected intraperitoneally (i.p.) with 10^4^*Trypanosoma cruzi* Colombiana parasites expressing nanoluciferase. ② Lamina propria cells were isolated from the colon of both infected and naive mice. ③ Cells from three infected and three naive mice were pooled and captured using the BD Rhapsody™ Single-Cell Analysis System. ④ After mRNA isolation and cDNA synthesis, libraries were prepared using the BD Rhapsody™ Whole Transcriptome Analysis Kit, and pooled libraries were sequenced on a NovaSeq 6000 sequencer. ⑤ Sequences were aligned to the mouse and *Trypanosoma cruzi* genomes. Single cells were clustered and annotated into distinct cell populations using cluster-specific markers and curated databases. ⑥ Cell-to-cell communication analysis was performed using CellChat.^21^ (B) Uniform manifold approximation and projection (UMAP) plot depicts 6,531 cells from naive mice (left panel) and 9,646 cells from chronically infected mice (right panel), with each point representing a single cell. ∗: enterocytes, enteroendocrine and smooth muscle cells; §: this population also includes two minor enterocyte clusters.

At 110 days post-infection, we dissected the colon tissue and confirmed the chronic infection status by *ex vivo* bioluminescence imaging (Figure S1). Single-cell suspensions were prepared from the LP of both naive and infected animals, using three mice per group. These suspensions were pooled, resulting in two samples that were processed separately following the BD Rhapsody™ Single-Cell Analysis System (BD Biosciences) workflow.^20^ The libraries generated from each pooled sample were combined and subjected to high-throughput sequencing (Figure 1A). Sequencing yielded high coverage across both samples, with a mean of 100,569 reads per cell and 7,684 unique molecular identifiers (UMIs) per cell in the chronically infected sample (11,500 cells), and 55,719 reads per cell and 5,837 UMIs per cell in the naive sample (9,500 cells). After QC filtering and alignment to the combined mouse-*T. cruzi* reference genome, 6,531 naive and 9,642 infected cells were retained for downstream analysis.

**Figure 2:**
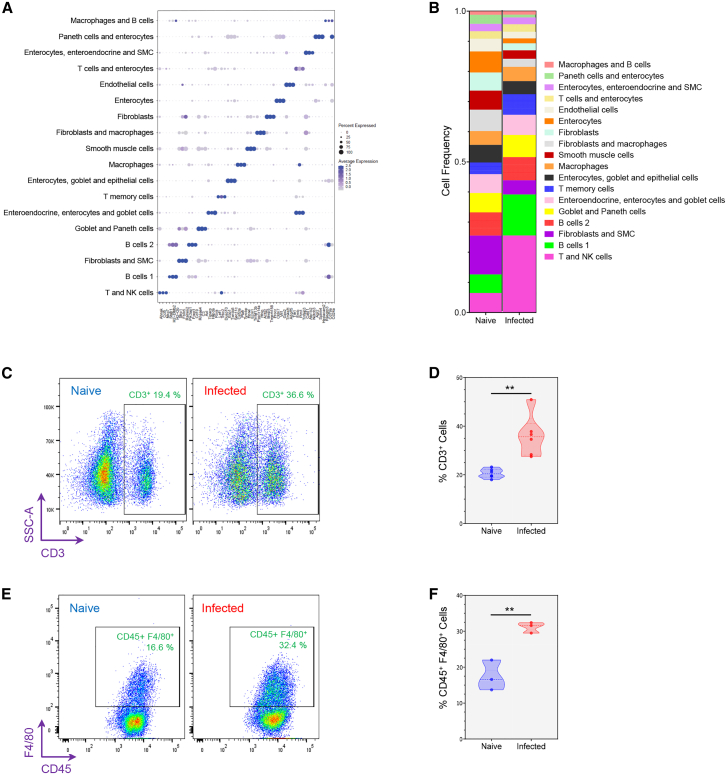
Flow cytometry analysis confirms the infiltration of T and NK cells and macrophages in the colon of *T. cruzi* chronically infected mice (A) Expression levels of the top three cluster-specific genes used to annotate cell populations. Dot size represents the percentage of cells expressing each gene, while dot color intensity indicates the expression level. (B) Bar graph shows the relative percentages of each cell type detected in naive and infected mice. (C) Representative flow cytometry plots show CD3^+^ cells in naive (left) and infected mice (right). (D) Violin plot displays the percentages of CD3^+^ cells in naive and infected mice. Data are representative of two independent experiments with a total of six mice per experiment. (E) Representative flow cytometry plots of CD45^+^ F4/80^+^ macrophages in naive (left) and infected mice (right). (F) Violin plot shows the percentages of CD45^+^ F4/80^+^ macrophages in naive and infected mice. Data are representative of two independent experiments with a total of 3 mice per group. Cells were gated on live, singlets, CD3^+^ or CD45^+^ F4/80^+^. In (D and F), violin plots display the distribution of values for each group. Individual points represent biological replicates. Large dashed horizontal lines indicate the median. Statistical significance was determined by an unpaired *t* test. ∗∗: *p* < 0.01.

Unsupervised clustering was performed to group cells, which were subsequently annotated into known cell populations based on cluster-specific markers and reference databases as detailed in the STAR Methods section.^22^ Clustering analysis revealed eighteen distinct cell clusters: T and NK cells; B cells (clusters 1 and 2); fibroblasts and smooth muscle cells; goblet and Paneth cells; enteroendocrine, enterocytes and goblet cells; T memory cells; enterocytes; goblet cells and epithelial cells; macrophages; fibroblasts and macrophages; smooth muscle cells; fibroblasts; enterocytes, endothelial cells; Paneth cells and enterocytes and macrophages and B cells (Figures 1B and 2B). A dot plot representing cell type-specific markers that were used to annotate each cell cluster is shown in Figure 2A. Consistent with previous findings,^18^ the uniform manifold approximation and projections (UMAPs) plots showed a significant recruitment of immune cells to the infected colon, including T and NK cells, B cells (cluster 1), and T memory cells, evidenced by increases in cell frequencies of 3.9-fold, 2.3-fold, and 1.7-fold, respectively, compared to naive controls (Figure 2B).

To confirm these findings—and throughout this work—we conducted independent experiments in which we prepared single-cell suspensions from the LP and performed flow cytometry analysis with cell-specific antibody panels as outlined in Figure S2. The results showed an increase in the percentage of CD3^+^ cells, rising from 19.4% in naive mice to 36.6% in chronically infected mice (Figures 2C and 2D). Likewise, we observed an expansion of macrophages (CD45^+^ F4/80^+^ cells), from 16.6% in naive mice to 32.4% in chronically infected mice (Figures 2E and 2F). In contrast, macrophage frequencies in the scRNA-seq dataset showed only a slight change and did not mirror the magnitude of expansion detected by flow cytometry (Figure 2B).

To identify specific colonic cell types that harbor *T. cruzi* parasites, we investigated the presence of parasitized cells in the scRNA-seq dataset. Our analysis revealed three heavily infected macrophages, with over 60% of the total RNA transcript content originating from the parasite (Figures S1B and S1C). This high proportion of parasite-derived transcripts indicated a significant infection burden within these cells. Among the *T. cruzi* transcripts detected, TcG-07726—encoding a mucin-associated surface protein (MASP)—was identified as the top-expressed gene. This protein is crucial for the parasite’s survival and plays a key role in evading the host immune response.^23^

These findings suggest that chronic *T. cruzi* infection is associated with reshaping of the colonic immune landscape, marked by a robust recruitment and expansion of both innate and adaptive immune cells.

### CD8^+^ cytotoxic T cells are the predominant subset within the T and NK cell compartment in the chronically infected colon

**Figure 3:**
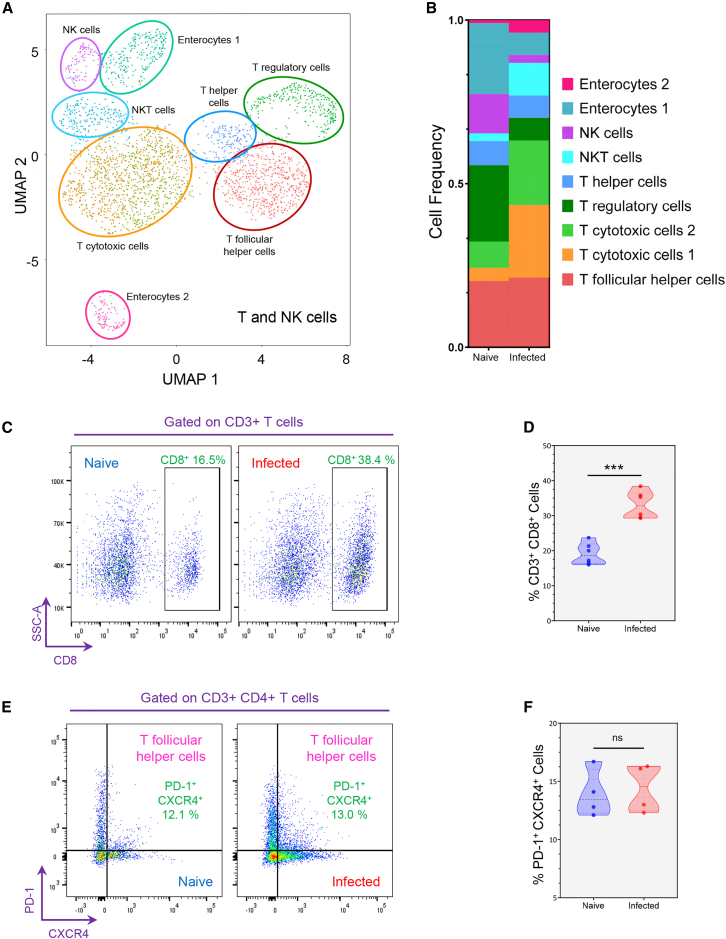
CD8^+^ T cytotoxic cells are the predominant subset within the T and NK cell compartment in the colon of chronically infected mice (A) Uniform manifold approximation and projection (UMAP) plot showing nine T and NK cell subclusters. Each point represents a single cell. (B) Bar graph depicts the relative percentages of each cell subtype in naive and infected mice. (C) Representative flow cytometry plots of CD8^+^ T cells in naive (left) and infected mice (right). Cells were gated on live, singlets, CD3^+^ cells. (D) Violin plot shows the percentage of CD8^+^ T cells in naive and infected mice. (E) Representative flow cytometry plots of T follicular helper cells in naive (left) and infected mice (right). Cells were gated on live, singlets, CD3^+^ CD4^+^ cells. (F) Violin plot shows the percentage of PD-1^+^ CXCR4^+^ T follicular helper cells in naive and chronically infected mice. Data are representative of two independent experiments with a total of 6 (in D) or 4 (in F) mice per group. In (D and F), violin plots show the distribution of values for each group. Individual points represent biological replicates. Large dashed horizontal lines indicate the median. Statistical significance was determined by an unpaired *t* test. ∗∗∗: *p* < 0.001; ns: not significant.

To investigate the diversity of T cell subtypes infiltrating the colonic LP, we performed refined sub-clustering of the major T and NK cell population. This analysis revealed subsets of T cytotoxic, T regulatory, T helper, T follicular helper, NK, and NKT cells. Additionally, two subclusters identified as enterocytes were spatially separated from the main T and NK cell population (Figure 3A).

A dot plot representing cell type-specific signature genes that were used to annotate cell subtypes is displayed in Figure S3. The T cytotoxic cell cluster consisted of two closely related cell types, both expressing *Cd8a*, *Cd8b1*, granzyme B (*Gzmb*, a gene associated with cytotoxic activity), and C–C chemokine receptor type 5 (*Ccr5*). Therefore, these cells were identified as CD8^+^ cytotoxic T cells. The two subclusters were subsequently consolidated into a single cluster for downstream analysis. A 1.9-fold increase in the frequency of the CD8^+^ cytotoxic T cell population was observed in the colon of chronically infected mice (Figure 3B). Flow cytometry analysis of LP single cell suspensions confirmed that the proportion of this population rose from 16.5% in naive mice to 38.4% in infected mice (Figures 3C and 3D). Follicular helper (Tfh) cells-defined by canonical markers *Cd4*, C-X-C chemokine receptor type 4 (*Cxcr4*), CD40 ligand (*Cd40L*), and interleukin-21 (*Il-21*)- were further identified by immunophenotyping with programmed cell death protein 1 (PD-1) and CXCR4 antibodies (Figures 3E and 3F). T regulatory (Treg) cells were distinguished by the expression of forkhead box P3 (*Foxp3*), Helios (*Ikzf2*), and cytotoxic T-lymphocyte antigen 4 (*Ctla-4*). T helper (Th) cells were identified by the expression of inducible T cell co-stimulator (*Icos*) and tumor necrosis factor (*Tnf*) ligand superfamily 8 (*Tnfsf8*), both associated with Th cell differentiation and activation. The NKT cell population, spatially positioned near the CD8^+^ cytotoxic T cell clusters, was defined by co-expression of *Cd3e, Cd8a*, killer cell lectin-like receptor E1 (*Klre1*), killer cell lectin-like receptor C1 (*Klrc1*), and *Gzmb*. In contrast, a small cluster of NK cells was distinguished by the expression of NK-specific markers such as *Klre1, Klrc1,* and *Gzmb*. Among these cell populations, it is noteworthy that the frequencies of Treg (7.5%) and NK cells (2.5%) were markedly reduced in the chronically infected colonic tissue compared to naive mice [Figure 3B; Treg cells (25%) and NK cells (13%)]. Together, these results suggest that *T. cruzi* infection drives a shift in the composition of colonic T and NK cell subsets, with a marked prevalence of CD8^+^ cytotoxic T cells.

### The CCL (C-C motif chemokine ligand) signaling pathway is likely activated in the mouse colon during chronic *T. cruzi* infection

**Figure 4:**
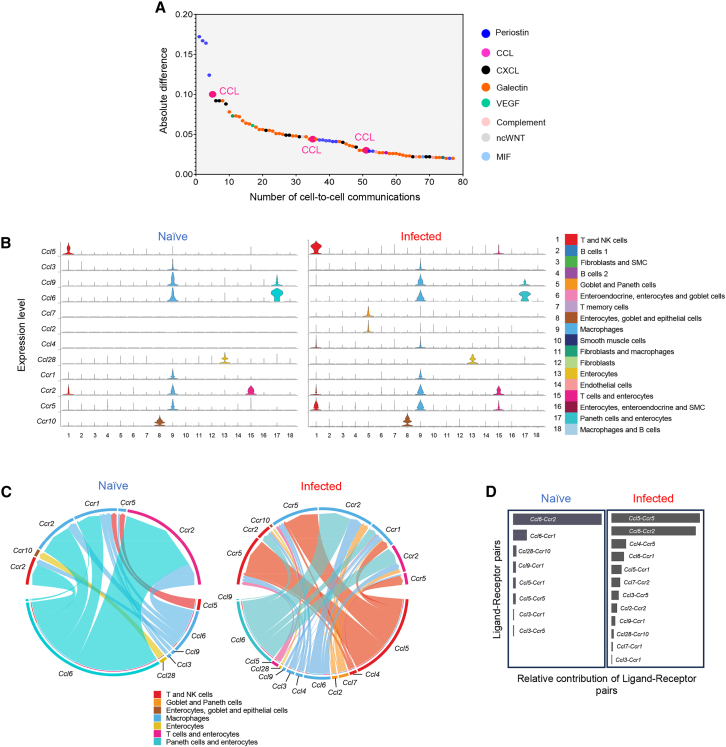
Predicted activation of the CCL signaling pathway network in the mouse colon during chronic *T. cruzi* infection (A) Rank-ordered communication probabilities for ligand-receptor interactions. Each point represents a specific ligand-receptor interaction, color-coded by signaling pathway as indicated in the legend. The CCL signaling pathway is highlighted in pink and labeled “CCL.” Data are plotted in descending order of communication probability. (B) Violin plots show the expression patterns of genes involved in CCL signaling in the colons of naive (left) and infected (right) mice. The y axis shows the expression levels of individual ligands or receptors, while the x axis denotes the corresponding cell populations. The width of each violin at a given expression level indicates the proportion of cells expressing that ligand or receptor. (C) Chord diagram illustrates inferred ligand–receptor interactions. Each segment represents a distinct cell population, with curved bands indicating the direction of signaling from sender to receiver cells. Band thickness does not reflect interaction strength. (D) Bar plot shows the predicted strength of communication for individual ligand–receptor pairs. The y axis lists specific ligand–receptor pairs, and the x axis indicates their relative contributions to the overall signaling activity. Contributions were estimated based on gene expression levels, network centrality, and interaction probabilities. All interaction data and communication scores were obtained using CellChat analysis of scRNA-seq information.^21^

Through our analysis, we identified several distinct immune cell types within the colonic tissue, providing insights into the local immune microenvironment in the context of chronic DCD. To gain deeper insight into potential intercellular signaling networks among these cells, we performed cell-cell communication analysis using CellChat,^21^ based on curated ligand-receptor interaction databases (Tables S1, S2, S3, and S4). Communication probabilities were inferred from ligand expression in sender cells and receptor expression in receiver cells (Tables S1 and S2). Using these predicted probabilities, we estimated the differences between chronically infected and naive samples (Table S3, “absolute difference” column). Out of approximately 2,000 predicted ligand-receptor interactions, significant communication events were identified by applying a 2% cutoff based on the observed absolute differences (Table S4). This analysis suggested multiple signaling pathways, including the CCL, complement, colony-stimulating factor (CSF), C-X-C motif chemokine ligand (CXCL), galectin, macrophage migration inhibitory factor (MIF), and periostin pathways. Among these networks, CCL signaling was predicted to be a key mediator of immune cell-cell communication as illustrated in Figure 4A, where absolute communication probabilities are plotted to highlight their relative significance. Other signaling networks identified in the analysis predominantly involved interactions between non-immune and immune cell populations and will be explored in future studies.

Violin plots were generated from single-cell RNA sequencing data to visualize the expression patterns of key ligand-receptor pairs. This analysis indicated a marked upregulation of *Ccl5* and its receptor *Ccr5* in the colonic tissue of chronically infected mice (Figure 4B, right panel). Particularly, the data suggested that T and NK cells, and macrophages were the predominant contributors to the CCL signaling axis, with T cells and enterocytes also participating, though to a lesser extent (Tables S3 and S4).

During chronic infection, T and NK cells were predicted to be key sender cells, secreting CCL5 that may signal both toward macrophages (receiver cells) and in an autocrine fashion, reinforcing their own activation. This directional signaling pattern is illustrated by the chord diagram depicted in Figure 4C (right panel), which shows the flow of ligand-receptor interactions among cell types. In contrast, naive mice exhibited a markedly different CCL signaling profile, with macrophages emerging as the predicted primary sender cells secreting CCL6 (Figure 4C, left panel). A total of 12 ligand–receptor pairs were identified in infected mice (Figure 4D, right panel), compared to 8 pairs in naive mice (Figure 4D, left panel). The CCL5-CCR5 axis is predicted to mediate signaling in the infected group, whereas the CCL6-CCR2 axis was the main signaling mechanism in naive mice.

**Figure 5:**
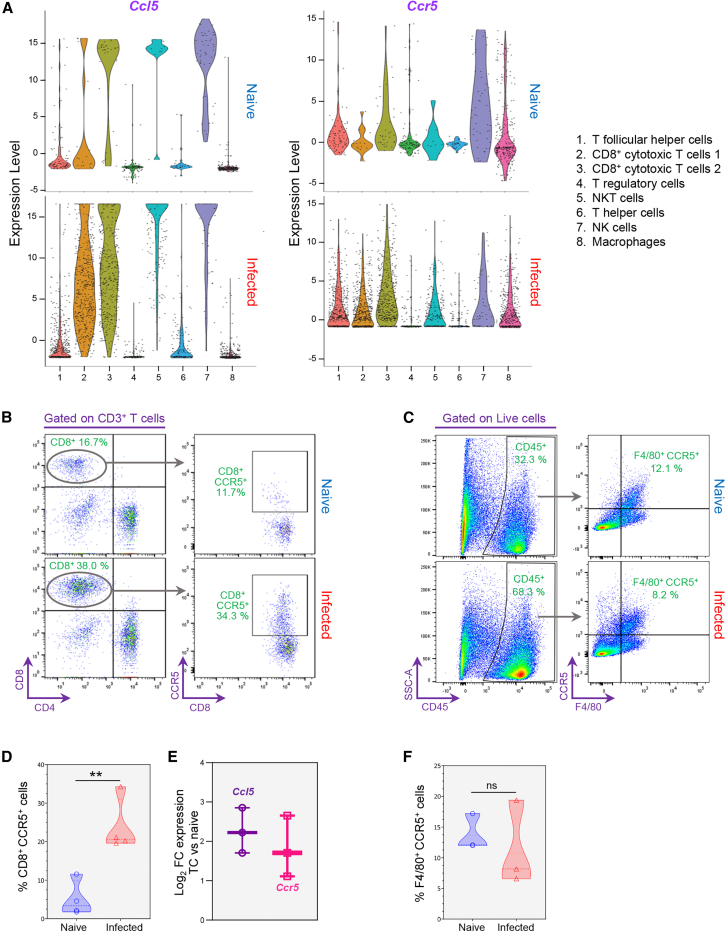
CD8^+^ cytotoxic T cells contribute as central mediators within the CCL signaling network (A) Violin plots show the distribution and density of CCL5 (left) and CCR5 (right) expression across T and NK cell subsets, as well as macrophages, in the colon of naive and chronically infected mice. Wider sections of the violin indicate a higher proportion of cells expressing CCL5 or CCR5 at that level. Data were generated from CellChat-guided analysis of scRNA-seq profiles.^21^ (B) Representative flow cytometry plots show CD8^+^ CCR5^+^ T cells in the colon of naive (upper panel) and chronically infected mice (bottom panel). Cells were gated on live, singlets, and CD3^+^ cells. (C) Representative flow cytometry plots show F4/80^+^ CCR5^+^ T cells in naive (upper panel) and chronically infected mice (bottom panel). Cells were gated on live, singlets, and CD45^+^ cells. (D) Violin plot shows the frequency of CD8^+^ CCR5^+^ T cells in naive and chronically infected mice. Data are representative of two independent experiments (*n* = 4 mice per group). (E) mRNA quantification of *Ccl5* and *Ccr5* by qRT-PCR in naive and chronically infected mice. The plot shows log_2_ fold changes (FC) in gene expression determined by the ΔC_t_ method with normalization to GAPDH. The horizontal line indicates the median value for each group, with individual data points plotted. CD8^+^ T cells were purified from the lamina propria of naive and chronically infected mice using magnetic bead–based separation prior to RNA extraction. Data are from three independent experiments. (F) Violin plot shows the frequency of F4/80^+^ CCR5^+^ T cells in the colon of naive and chronically infected mice. Data are representative of two independent experiments (*n* = 3 mice per group). In (D and F), violin plots show the distribution of values for each group. Individual points represent biological replicates. Large dashed horizontal lines indicate the median. Statistical significance was determined by an unpaired *t* test. ∗∗: *p* < 0.01; ns: not significant.

Comparison of *Ccl5* and *Ccr5* expression across T and NK immune cell subtypes in infected versus naive mice revealed a significant increase in the expression of both genes in the two CD8^+^ cytotoxic T cell subclusters (Figures 5A and 5B, left panels). In contrast, macrophages showed only background-level expression of *Ccl5* regardless of infection status (Figure 5A, left panels), while *Ccr5* expression was moderately upregulated in chronically infected mice (Figure 5A, right panels). Flow cytometric analysis, conducted on LP cells from an independent experiment, confirmed the upregulation of CCR5 expression. The percentage of CD8^+^CCR5^+^ among CD3^+^ T cells increased from 11.7% in naive mice to 34.3% in chronically infected mice (Figures 5C and 5D). Moreover, consistent with the transcriptomic data, quantitative reverse transcription PCR (qRT-PCR) demonstrated a 7-fold increase in *Ccl5* and a 2-fold increase in *Ccr5* transcript levels in LP cells isolated from infected mice compared to naive controls (Figure 5E).

Our observations collectively strongly suggest that chronic *T. cruzi* infection leads to the activation of CCL-mediated immune signaling within the colon, with CD8^+^ cytotoxic T lymphocytes probably acting as primary drivers of this process. The CCL5-CCR5 axis is likely to play a prominent role, indicative of a highly coordinated network of immune cell communication. This highlights a dynamic and localized immune response in the mouse colon that may be critical for maintaining effective tissue surveillance. These findings underscore the importance of chemokine-driven signaling in shaping the colonic immune microenvironment, revealing potential mechanisms of parasite persistence.

### CD8^+^ cytotoxic T cell-macrophage interactions contribute to colonic immune homeostasis during chronic *T. cruzi* infection

Guided by the cell–cell communication analysis, which predicted CD8^+^ cytotoxic T cells and macrophages as major signaling hubs, we focused our differential gene expression (DGE) analysis on these cell populations. To link transcriptional changes to the observed intercellular signaling events, we compared the RNA profiles of CD8^+^ cytotoxic T cells and macrophages from chronically infected versus naive mice (Tables S2 and S3). This approach enabled the identification of immune cell–specific gene expression signatures associated with chronic infection.

**Figure 6:**
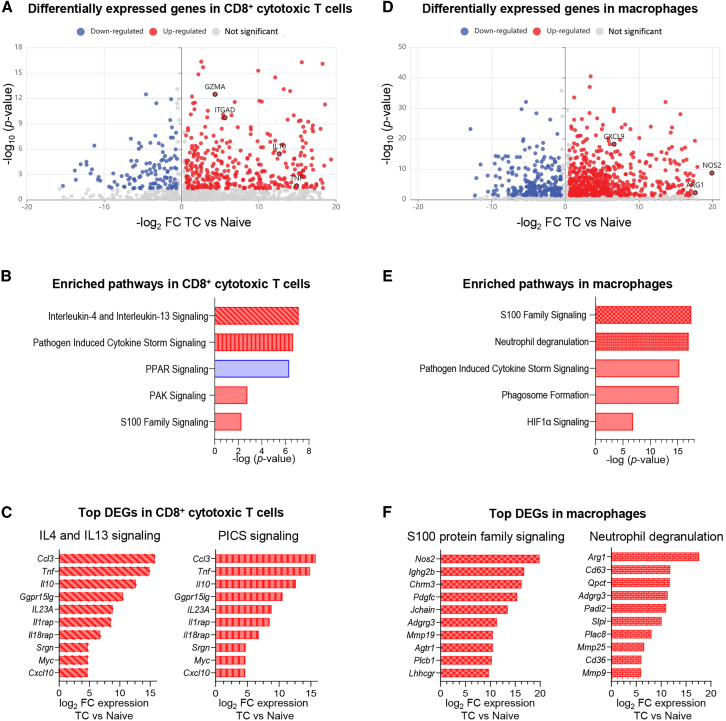
CD8^+^ cytotoxic T cell–macrophage interactions likely shape colonic immune homeostasis during chronic *T. cruzi* infection (A) Volcano plot showing differentially expressed genes (DEGs) in lamina propria CD8^+^ cytotoxic T cells from infected mice compared with naive controls. Red dots depict upregulated genes, blue dots indicate downregulated genes, and gray dots represent genes with a non-significant change in expression. Genes with log_2_ fold change ≥1.5, and *p* < 0.05 were considered significant. (B) Top five differentially regulated canonical pathways in lamina propria CD8^+^ cytotoxic T cells from infected mice, as predicted by ingenuity pathway analysis (IPA). Bars represent −log (*p* value); red indicates pathway activation and blue indicates pathway inhibition. (C) Top 10 upregulated genes associated with the IL-4/IL-13 signaling pathway (left) and the pathogen-induced cytokine storm (PICS) pathway (right) in CD8^+^ cytotoxic T cells. (D) Volcano plot of DEGs in lamina propria macrophages from infected mice compared with naive controls, displayed as in (A). (E) Top five differentially regulated canonical pathways in lamina propria macrophages from infected mice, as predicted by IPA, displayed as in (B). (F) Top 10 upregulated genes associated with the S100 protein family signaling pathway (left) and the neutrophil degranulation pathway (right) in macrophages. In (C and F), bars indicate log_2_ fold change between infected and naive mice.

**Figure 7:**
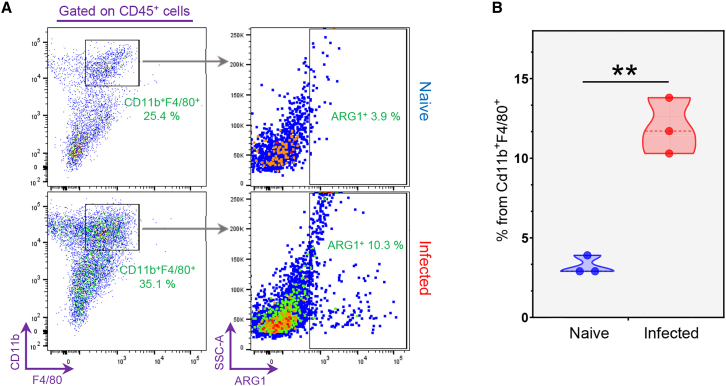
Increased frequency of ARG1^+^ macrophages in the colon during chronic *T. cruzi* infection (A) Representative flow cytometry analysis of arginase-1 (ARG1) expression in colonic macrophages from naive and infected mice. Cells were gated on live, singlets, and CD45^+^ cells, followed by identification of CD11b^+^F4/80^+^ macrophages. ARG1 expression was assessed within the CD11b^+^F4/80^+^ population in naive (upper panels) and infected (lower panels) mice. (B) Violin plot shows the frequency of ARG1^+^ CD11b^+^F4/80^+^ cells. Data are representative of two independent experiments (*n* = 3 mice per group). Violin plots show the distribution of values for each group. Individual points represent biological replicates. Large dashed horizontal lines indicate the median. Statistical significance was determined using an unpaired *t* test. ∗∗: *p* < 0.01.

The CD8^+^ cytotoxic T cells cluster from infected mice was distinguished by the upregulation of several key transcripts, including interleukin-10 (*Il10*), *Tnf, Gzma,* and integrin alpha-D (*Itgad*), which are associated with immunomodulation, cytotoxic function, and cell adhesion (Figure 6A; Table S5). Macrophages likewise exhibited a distinct transcriptional profile consistent with an activated state following parasite infection (Figure 6D; Table S6). We detected elevated expression of C-X-C motif chemokine ligand 9 (*Cxcl9*), nitric oxide synthase 2 (*Nos2*), and arginase 1 (*Arg1*) —genes associated with inflammatory signaling and cellular metabolism. Upregulation of ARG1 was confirmed by flow cytometry in macrophages (Figure 7). Moreover, expression of all three immune-related genes (*Arg1, Cxcl9, and Nos2*) was validated by qRT-PCR (Figure S4), supporting the robustness of the transcriptomic analysis.

To study the signaling pathways activated in response to *T. cruzi*, we analyzed differentially expressed genes using Ingenuity Pathway Analysis (IPA).^24^ In CD8^+^ T lymphocytes, the top enriched canonical pathways included interleukin-4 (IL-4) and interleukin-13 (IL-13) signaling, pathogen-induced cytokine storm (PICS), p21-activated kinase (PAK) and S100 protein family signaling (Figure 6B). We further examined the gene expression profile of molecules within the IL-4 and IL-13 signaling pathway (Figure 6C, left panel), known to contribute to alternative (M2) macrophage polarization.^25^ Notably, the most up-regulated genes were *Tnf*, intercellular adhesion molecule 1 (*Icam1*), integrin alpha-X (*Itgax*), vimentin (*Vim*), and *Il10*. In the context of the PICS signaling pathway (Figure 6C, right panel)—a process characterized by excessive cytokine production and uncontrolled inflammation^26^—we also observed significant upregulation of *Tnf* and *Il10*, along with other key genes such as *Ccl3*, G protein–coupled receptor 15 ligand (*Gpr15lg*), and interleukin-23 subunit alpha (*Il23a*).

Among macrophages, the most enriched canonical pathways included S100 protein family signaling, neutrophil degranulation, PICS signaling, phagosome formation, and hypoxia-inducible factor 1-alpha (HIF1α) signaling pathways (Figure 6E). We next studied the gene expression profile of molecules associated with the S100 protein family signaling pathway (Figure 6F, left panel) - a group of calcium-binding proteins that are activators of immune functions such as inflammation and play an important role in calcium homeostasis.^27^ The most up-regulated genes comprised *Nos2* [a hallmark of classically activated (M1) macrophages^28^^,^^29^], immunoglobulin heavy constant gamma 2B (*Ighg2b*), cholinergic receptor muscarinic 3 (*Chrm3*), platelet-derived growth factor C (*Pdgfc*) and joining chain of multimeric IgA and IgM (*Jchain*). Within the neutrophil degranulation pathway—a process characterized by the release of antimicrobial granules^30^—we observed upregulation of genes such as *Arg1* [a protein typically associated with alternatively activated macrophages (M2) macrophages^28^^,^^29^], C*d63*, glutaminyl-peptide cyclotransferase (*Qpct*), adhesion G protein–coupled receptor G3 (*Adgrg3*), and peptidyl arginine deiminase 2 (*Padi2*) (Figure 6F, right panel).

Other T cell populations were also predicted to contribute to M2 macrophage polarization (Figure S5). Specifically, our analysis suggests that Th and Tfh cells express *Il4* and the transcription factor GATA-binding protein 3 (*Gata3*), as observed in the UMAP projections from our scRNA-seq data. GATA3 is a key transcription factor required for IL-4 synthesis and Th2 cell differentiation.^31^ In addition, we detected *Il10* expression in Treg, Tfh, and CD8^+^ cytotoxic T cells. IL-10 is known to modulate macrophage activation by suppressing pro-inflammatory responses and promoting anti-inflammatory, M2-associated gene expression, and functions.^32^ Collectively, these predictions, based on gene expression patterns, highlight potential cellular sources of M2-polarizing signals during chronic *T. cruzi* infection and warrant further experimental validation.

In summary, chronic *T. cruzi* infection likely elicits coordinated transcriptional programs in CD8^+^ cytotoxic T cells and macrophages, balancing pro-inflammatory responses—evidenced by *Ifng, Tnf*, *Cxcl9*, and *Ccl5* expression—with regulatory mechanisms such as *Il10* and *Il4* production. These findings support a model in which CD8^+^ cytotoxic T cells may drive autocrine and paracrine CCL5-CCR5 signaling to amplify effector functions while simultaneously modulating local immune responses, thereby potentially promoting both parasite control and tissue protection.

## Discussion

Although less studied than the cardiac form, approximately 5–15% of infected individuals develop DCD, which has a substantial health impact, particularly in endemic regions.^5^^,^^33^ The colon is a major site of *T. cruzi* persistence,^10^^,^^11^ where enteric neuropathy can lead to progressive disorders such as megacolon and impaired motility.^8^ Understanding the immunological landscape within the colonic tissue during chronic infection is therefore critical for revealing the mechanisms involved in both parasite long-term persistence and tissue pathology.

Our work provides a comprehensive single-cell transcriptomic analysis of the colonic LP immune cells, expanding our understanding of the host response to the parasite within this tissue. Through single-cell RNA sequencing, we achieved high-resolution identification of distinct immune cell subsets, their activation states, and transcriptional programs. Building on these data, computational inference of cell–cell communication enabled the dissection of intercellular signaling networks, together providing an integrated view of the immune milieu and the complex cellular interactions predicted to modulate tissue-specific defense mechanisms.

We found that chronic infection results in a reshaping of the colonic LP cellular heterogeneity, marked by the infiltration of T and NK cells, B cells, macrophages, and T memory cells (Figures 1B and 2B). Among the T and NK cell subsets, CD8^+^ cytotoxic T cells were markedly enriched (Figures 3A and 3B), suggesting a role as key mediators of local immune activation, in line with previous studies conducted across different tissues known for parasite persistence.^18^^,^^34^^,^^35^ Other T cell subclusters included CD4^+^ T cells, such as Tfh cells and Treg cells. While the frequency of Tfh cells remained almost unchanged in infected mice compared to naive controls, single-cell transcriptomic analysis indicated a relative depletion of Treg cells and NK cells (Figures 3B and 3D). Treg cells are essential for maintaining a balanced immune response and preventing excessive pathology.^36^ This reduction may contribute to the maintenance of continuous protective CD8^+^ T cell immunity^37^; however, functional validation at the protein level was not performed, and these findings should be interpreted as reflecting transcriptional trends rather than direct measurements of cell numbers or function.

Although macrophages were consistently detected in the LP, their relative abundance in the scRNA-seq dataset showed only a slight change during chronic infection compared with the expansion observed by flow cytometry (Figures 2B, 2E, and 2F). This discrepancy likely reflects technical differences between cell isolation approaches, as the Percoll-based enrichment used for scRNA-seq may underrepresent highly adherent or extracellular matrix–associated macrophage subsets.^38^ Nevertheless, both approaches consistently detected macrophages, confirming their robust presence in the LP of chronically infected mice.

The immune cell composition changes observed in the chronically infected colon prompted us to explore, through predictive cell-to-cell communication analysis, the potential contribution of intercellular signaling to the modulation of the local immune response. Using CellChat analysis, we predicted the activation of the CCL signaling pathway—particularly the CCL5–CCR5 axis—as a major intercellular communication mechanism (Figure 4). CD8^+^ cytotoxic T cells were the predominant source of CCL5 and exhibited significantly elevated CCR5 expression, suggesting autocrine and paracrine signaling loops that could amplify their effector functions during chronic infection.

In contrast, macrophages did not secrete CCL5 and primarily functioned as signal receivers, expressing lower levels of CCR5 compared to CD8^+^ cytotoxic T cells, consistent with their predicted role as target cells (Figures 4B, 5A and 5C). These observations are particularly relevant given that cytokines and chemokines are well-established modulators of immune mechanisms that not only mitigate tissue damage but also influence disease progression.^35^ CCL5 secreted by CD8^+^ cytotoxic T cells is likely to act as a potent chemoattractant, promoting macrophage recruitment and activation.

This inferred interaction underscores the importance of the CCL5-CCR5 signaling axis. Its role in intestinal inflammation is supported by extensive evidence from both CD and other inflammatory gut pathologies. Pre-clinical models of DCD have demonstrated a direct upregulation of *Ccl5* expression within the chronically inflamed colon, linking this chemokine to the local Type 1 inflammatory response responsible for neuronal destruction.^13^^,^^39^ Histological studies indicate elevated CCL5 in inflamed mucosal regions, with CCR5 expressed on neighboring immune cells, highlighting the axis’s role in coordinating inflammatory cell recruitment in inflammatory bowel disease (IBD) tissue.^40^^,^^41^ Further, spatial transcriptomic analyses have demonstrated that immune responses are organized in spatially restricted niches that promote T cell recruitment and localization,^42^ with the CCL5–CCR5 axis playing a key role in driving inflammatory cell recruitment and activation in IBD.^43^ Together, these findings provide histopathological, functional, and spatial support for a biologically active role of the CCL5–CCR5 axis in mediating chronic intestinal inflammation, aligning with our transcriptomic predictions.

Consistent with previous studies,^17^^,^^44^ our analysis suggests that CD8^+^ cytotoxic T cells exhibit a strong effector phenotype based by the expression of genes such as *Ccl5*, *Ccr5,* Gzma*, Tnf*, and *Infg* (Figures 5, 6A, S4, and S5). In addition, qRT-PCR results (Figure S4), together with a bioinformatic analysis (Figure S5), support the possibility that these cells may express *Il10*, indicating a potential immunoregulatory role. In chronic Chagas disease, IL-10 helps balance parasite control while limiting heart tissue damage.^45^ Although it is well known for maintaining immune homeostasis in the GI tract,^46^ its expression by CD8^+^ cytotoxic T cells in DCD still requires further experimental confirmation.

In addition to classical effector markers, CD8^+^ cytotoxic T cells showed the activation of the IL-4/IL-13 and PICS signaling pathways. While IL-4/IL-13 signaling is typically associated with type 2 immune responses and is known to modulate inflammation, tissue repair, and fibrosis,^47^ the activation of the PICS network is linked to uncontrolled release of pro-inflammatory cytokines.^26^^,^^48^ The simultaneous enrichment of regulatory- and pro-inflammatory–associated gene programs highlights the transcriptional complexity of colonic CD8^+^ cytotoxic T cells during chronic *T. cruzi* infection. Functional validation of these pathways at the protein and cellular levels will be the focus of future studies.

After entering host cells, trypomastigotes are phagocytosed by macrophages, which can polarize along a functional spectrum between two major activation states: M1 (classically activated), associated with pro-inflammatory and microbicidal functions, and M2 (alternatively activated), which promotes tissue repair and immunoregulation.^29^ Our macrophage transcriptional profiling uncovered key insights into their activation and functionality.

We observed approximately a 2-fold increase in macrophage frequency (Figures 2B, 2E, and 2F). Pathways analysis predicted significant enrichment of neutrophil degranulation and S100 protein family signaling, alongside upregulation of *Nos2* and *Arg1* (Figures 6B, 6D, 7, and S4). Interestingly, NOS2, a hallmark of M1 activation, is responsible for nitric oxide production—a key antimicrobial molecule involved in parasite killing.^28^ In contrast, ARG1, associated with M2 activation, contributes to polyamine synthesis, and facilitates parasite replication and survival within macrophages.^28^^,^^49^

These results predict the concurrent presence of M1 and M2 macrophages and highlight their functional plasticity in modulating immune responses based on local environment cues. Since NOS2 and ARG1 compete for the same substrate, L-arginine, the balance between these enzymes may influence whether parasites are eliminated or persist in the colon. It is important to note that the M1/M2 paradigm represents a simplification of a more complex and dynamic spectrum of macrophage activation, as discussed in recent literature.^50^^,^^51^ Our analysis relied on canonical markers, NOS2 and ARG1, to infer M1-and M2-like macrophages. While additional markers and functional assays could provide higher resolution of macrophage states, this framework serves as a well-established starting point to interpret the balance of inflammatory versus regulatory signals within the chronically infected colon.

Lastly, we identified three macrophages harboring high levels of parasite RNA spatially separated from the main macrophage cluster (Figures S1B and S1C); however, we could not determine whether they belonged to the M2 subtype.

**Figure 8:**
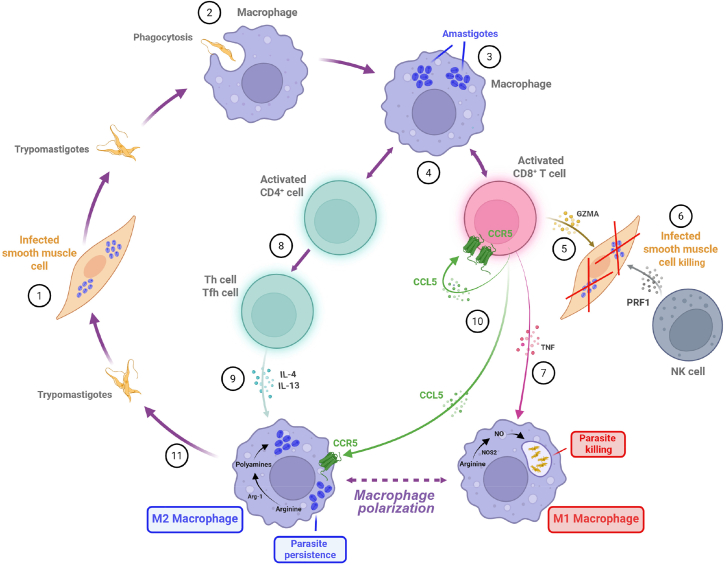
Proposed model of the immune response mechanisms to *Trypanosoma cruzi* in the mouse colon, integrating our findings with prior studies ① Infected smooth muscle cells release trypomastigotes into the surrounding colonic tissue. ② Trypomastigotes are phagocytosed by macrophages. ③ After 18–24 hs post-internalization, amastigotes are released into the cytoplasm, where they replicate and synthesize proteins that are presented on the macrophage surface. ④ CD8^+^ and CD4^+^ T cells recognize *T. cruzi* peptides presented by MHC complexes. ⑤ Upon activation, CD8^+^ T cells secrete granzyme A and multiple cytokines. ⑥ Granzyme A -containing granules may enter parasite-infected smooth muscle cells through pores formed by perforin-1 (PRF1; secreted by NK cells), contributing to cell death. ⑦ Tumor necrosis factor (TNF) may promote macrophage polarization to an M1 phenotype by inducing the expression of inducible nitric oxide synthase (NOS2). This enzyme subsequently oxidizes arginine to produce nitric oxide, which may contribute to parasite killing. ⑧ Activated CD4^+^ T cells can differentiate into Th and Tfh cells that produce Interleukin-4 (IL-4) and Interleukin-13 (IL-13) [⑨], which in turn induce M2 macrophage polarization. ⑩ C-C Motif Ligand 5 (CCL5) could further support M2 macrophage polarization via arginase I (ARG1) upregulation, promoting polyamines associated with parasite multiplication and persistence. ⑪ Amastigotes differentiate into trypomastigotes, completing the cycle by infecting naive smooth muscle cells.

As shown schematically in Figure 8, this work outlines a transcriptomics-driven model that integrates our data with prior studies to propose immunological pathways associated with either *T. cruzi* clearance or persistence in the chronically infected mouse colon. By integrating gene expression data with cell–cell communication analyses and existing literature, we propose a mechanistic framework for the host response to the parasite. The cycle begins with rupture of infected smooth muscle cells (step 1),^12^ releasing trypomastigotes that are subsequently phagocytosed by macrophages (step 2). Within macrophages, parasites differentiate into amastigotes and replicate (step 3).^52^ Infected macrophages then present parasite antigens, leading to the activation of CD4^+^ and CD8^+^ T cells (step 4).

Our transcriptomic data showed increased expression of genes such as *Gzma* (step 5) and *Prf1* (step 6) that support the prediction of a cytotoxic response driven by CD8^+^ T cells and NK cells, respectively. This leads to the elimination of infected cells, a finding consistent with previously documented mechanisms.^14^ At this stage of the immune response, multiple T cell subsets are predicted to concurrently shape macrophage functional states. Inferred CD8^+^ T-cell–associated signaling, including TNF-linked pathways, suggests a contribution to macrophage polarization toward a pro-inflammatory M1 phenotype (step 7). This activation state is associated with the induction of *Nos2,* with increased transcript abundance in our dataset, leading to the production of nitric oxide that contributes to intracellular parasite control.^49^ In parallel, CD4^+^ T helper–associated signaling pathways (step 8), including IL-4 and IL-13, are predicted to promote alternative (M2) macrophage polarization (step 9), supported by the elevated expression of *Arg1* as verified in this work (Figure 7). Established literature demonstrates that IL-4 and IL-13 are potent inducers of M2 polarization and that ARG1 competes with NOS2 for their common substrate, L-arginine, promoting parasite survival.^28^^,^^49^ Our cell–cell communication analysis predicts that the CCL5-CCR5 axis (step 10) acts as a critical coordinator of these divergent yet coexisting immune programs, likely by regulating the recruitment of effector T cells and macrophages within infected tissue. Finally, intracellular amastigotes within M2 macrophages can persist and subsequently differentiate into trypomastigotes, which, upon release (step 11), reinitiate infection of neighboring smooth muscle cells, thereby completing the cycle of chronic infection within colonic tissue. Importantly, while the figure simplifies spatial dynamics, immune cells and parasites may traffic between the LP and muscle layers, suggesting a compartmentalized yet interconnected immune response within colonic tissue.

Taken together, our findings highlight a chemokine-driven immune microenvironment in the colon during chronic *T. cruzi* infection. CD8^+^ cytotoxic T cells may act as key immune orchestrators, promoting localized tissue responses through their cytotoxic activity and chemokine-mediated recruitment of other immune cells. Macrophages, in turn, adopt both activated and permissive phenotypes, potentially contributing to a dynamic but incomplete containment of the parasite. Notably, we identified the CCL5–CCR5 axis as a central component of this immune network, suggesting its critical role in mediating cellular recruitment and immune signaling within the colon. Despite this coordinated immune response—and the ability of both macrophages^53^ and CD8^+^ T cells^18^ to infiltrate the colonic circular muscle layer, complete parasite clearance remains elusive. The potential contribution of *T. cruzi*–induced gut microbiota dysbiosis to disrupted mucosal immunity cannot be excluded. Previous studies suggest that chronic infection may alter microbial composition and microbial-derived metabolites, which could in turn influence pro- and anti-inflammatory cytokine production, as well as immune cell recruitment and function within the gut mucosa.^54^^,^^55^^,^^56^ Further research will be needed to dissect additional mechanisms underlying immune evasion, tissue-specific immune regulation, and the chronic persistence of *T. cruzi* within GI tissues. In this context, the identification of the CCL5-CCR5 axis as a central feature of the inflammatory network raises the possibility that therapeutic strategies targeting this pathway may help modulate pathogenic immune responses and preserve tissue function in DCD. Moreover, components of this signaling axis may serve as potential biomarkers of disease progression or local inflammatory activity within the GI tract. However, given evidence from experimental models of *T. cruzi* infection indicating that CCR5-mediated signaling also contributes to parasite control,^57^ careful evaluation of such approaches will be required to balance anti-inflammatory effects with host defense.

### Limitations of the study

Our study provides a comprehensive overview of the transcriptomic profiles and predicted cellular communication networks in the mouse colon during chronic *T. cruzi* infection. While these findings offer valuable insights into colonic immune cell responses, certain limitations should be considered when interpreting the data.

Pooling samples from three infected and three naive animals improved cell recovery and reduced technical variability; however, this approach limited our ability to assess inter-individual variability in immune cell composition and transcriptional heterogeneity.

Immune cell phenotypes and intercellular interactions were inferred primarily from transcriptomic and bioinformatics-based analyses. Although the inferred M1-and M2-like macrophage phenotypes were supported by canonical markers *Nos2*and*Arg1*, reliance on these markers alone limits the resolution of macrophage activation states and may not fully capture the dynamic spectrum of phenotypes present in the tissue. Further studies incorporating expanded surface marker profiling, functional assays, metabolomics, and spatial transcriptomics will be required to more fully define immune cell states and validate predicted cell–cell interactions.

Because only female mice were included, potential sex-dependent differences in immune responses during chronic *T. cruzi* infection were not assessed and could be addressed in future studies.

Finally, although the mouse model offers a controlled system to explore the mechanisms of CD pathogenesis, differences in immune architecture and disease progression between mice and humans may limit the direct translatability of the results.

## Resource availability

### Lead contact

Further information and requests for resources and reagents should be directed to and will be fulfilled by the lead contact, Erica Silberstein (erica.silberstein@fda.hhs.gov).

### Materials availability

This study did not generate new unique reagents.

### Data and code availability

• Single-cell RNA-seq data generated in this study are publicly available in Gene Expression Omnibus (GEO) under accession no. GSE319934.
• This paper does not report original code.
• Any additional information required to reanalyze the data reported in this paper is available from the lead contact upon request.

## Acknowledgments

This research was supported by intramural research funds from the U.S. Food and Drug Administration to A.D. We thank Dr. Sreenivas Gannavaram for his invaluable advice and thoughtful discussions; Laura Davis (FDA Division of Veterinary Services) as well as Adovi Akue and Mark Kukuruga (FDA CBER Flow Cytometry Core) for technical assistance; and Chloe Charendoff (BD Biosciences) for her expert guidance with the single-cell RNA sequencing workflow and libraries preparation. The graphical abstract and Figure 8 were created in https://BioRender.com. The findings and conclusions in this article have not been formally disseminated by the Food and Drug Administration and should not be construed to represent any Agency determination and policy.

## Author contributions

E.S.: conceptualization, formal analysis, investigation, methodology, validation, visualization, writing – original draft, and writing – review and editing.; S.K.: data curation, formal analysis, software, visualization, writing – original draft, and writing – review and editing; N.T.: data curation and formal analysis; C.C.C.: data curation and formal analysis; S.K.: methodology; J.S.C.: methodology; A.D.: conceptualization, supervision, and writing – review and editing.

## Declaration of interests

The authors declare no competing interests.

## Declaration of generative AI and AI-assisted technologies in the writing process

During the preparation of this work, the authors used ChatGPT to check grammatical issues and improve sentence structure. After using this tool, the authors reviewed and edited the content as needed and take full responsibility for the content of the publication.

## STAR★Methods

### Key resources table

**Table:** 

REAGENT or RESOURCE	SOURCE	IDENTIFIER
**Antibodies**		

anti-ARG1-PE	BioLegend	Cat-165804, RRID: AB_3068116
anti-CCR5-AF488	BioLegend	Cat-107008, RRID: AB_528756
anti-CD3-BV421	BioLegend	Cat-100228, RRID: AB_2562553
anti-CD3- PE-CF594	BD Biosciences	Cat-562280, RRID: AB_11153674
anti-CD4-APC	Miltenyi Biotec	Cat-130-123-207, RRID: AB_2811460
anti-CD4-PE	Miltenyi Biotec	Cat-130-116-509, RRID: AB_2727582
anti-CD45-FITC	BD Biosciences	Cat- 553080, RRID: AB_394610
anti-CD8-APC	Miltenyi Biotec	Cat-130-117-776, RRID: AB_2728039
anti-F4/80-BV421	BioLegend	Cat-123137; RRID: AB_2563102
anti-PD1-RB705	BD Biosciences	Cat- 570654, RRID: AB_3685931
anti-CXCR4-PE	BD Biosciences	Cat-561734, RRID: AB_11154227
Ghost Dye™ Violet 510 Fixable Viability Dye	Cell Signaling Technology	Cat-59863
BD Horizon™ Fixable Viability Stain 575V	BD Biosciences	Cat-565694

**Bacterial and virus strains**		

*Trypanosoma cruzi* (TcCOL-NLuc)	Silberstein et al., 2018	PMDI: 29672535

**Chemicals, peptides, and recombinant proteins**		

Percoll^TM^ density gradient media	Cytiva	Cat- 17089101
Calcein AM	Thermo Fisher Scientific	Cat-C1430
Draq7^TM^	BD Biosciences	Cat-564904

**Critical commercial assays**		

Agencourt® AMPure® XP magnetic beads	Beckman Coulter	Cat-A63880
BD Rhapsody^TM^ cartridge kit	BD Biosciences	Cat-633733
BD Rhapsody^TM^ cDNA kit	BD Biosciences	Cat-633773
BD Rhapsody^TM^ WTA amplification kit	BD Biosciences	Cat-633801
CD8a (Ly-2) Microbeads	Miltenyi Biotec	Cat-130-117-044
gentleMACS™ Dissociator	Miltenyi Biotec	Cat-130-093-235
gentleMACS™ C Tubes	Miltenyi Biotec	Cat- 130-093-237
iScript gDNA Clear cDNA Synthesis Kit	Bio-Rad Laboratories	Cat-1725034
Lamina Propria Dissociation Kit, mouse	Miltenyi Biotec	Cat-130-097-410
LS Columns	Miltenyi Biotec	Cat-130-042-401
Nano-Glo® Luciferase System	Promega	Cat-N1130
PureLink™ RNA Mini Kit	Thermo Fisher Scientific	Cat-12183020
Qubit dsDNA High Sensitivity (HS) Assay Kit	Thermo Fisher Scientific	Cat-Q32851
SsoAdvanced™ Universal SYBR® Green Supermix	Bio-Rad Laboratories	Cat-1725271
ViaStain™ AOPI staining solution	Revvity, Inc.	Cat- CS201065ML

**Deposited data**		

Raw and analyzed data from scRNA-seq	This paper	GSE19934

**Experimental models: Cell lines**		

LLC-MK2 cells	ATCC	Cat-CCL-7™

**Experimental models: Organisms/strains**		

C57BL/6J	Charles River Laboratories	*Mus Musculus*

**Oligonucleotides**		

ARG1	Bio-Rad Laboratories	ID: qMmuCID0022400
CCL5	Bio-Rad Laboratories	ID: qMmuCED0049026
CCR5	Bio-Rad Laboratories	ID: qMmuCID0020341
CXCL9	Bio-Rad Laboratories	ID: qMmuCID0023784
GAPDH	Bio-Rad Laboratories	ID: qMmuCED0027497
GZMA	Bio-Rad Laboratories	ID: qMmuCID0008918
IFNG	Bio-Rad Laboratories	ID: qMmuCID0006268
ITGAD	Bio-Rad Laboratories	ID: qMmuCID0014548
IL10	Bio-Rad Laboratories	ID: qMmuCED0044967
NOS2	Bio-Rad Laboratories	ID: qMmuCID0023087
TNF	Bio-Rad Laboratories	ID: qMmuCED0004141

**Software and algorithms**		

CellMarker	Zhang et al.^58^	http://biocc.hrbmu.edu.cn/CellMarker/
CellMarker v2.0	Hu et al.^59^	http://biocc.hrbmu.edu.cn/CellMarker/
DESeq2 v1.40.2	Love et al.^60^	https://bioconductor.org/packages/DESeq2
FlowJo v.10	BD Biosciences	https://www.flowjo.com/
Ingenuity Pathway Analysis (IPA)	Qiagen Inc.	https://www.qiagen.com/us/products/discovery-and-translational-research/next-generation-sequencing/informatics-and-data/interpretation-content-databases/ingenuity-pathway-analysis
PanglaoDB	Franzen et al.^61^	https://panglaodb.se
R v4.3.3	R Core Team^62^	https://cran.r-project.org/bin/windows/base/old/4.3.3/
R-package Seurat v4.4	Hao et al.^63^	https://satijalab.org/seurat/
R-package CellChat v1	Jin et al.^21^	https://github.com/sqjin/CellChat
scDblFinder v1.10	Germain et al.^64^	https://bioconductor.org/packages/scDblFinder/
Tabula Muris	The Tabula Muris Consortium^65^	https://tabula-muris.ds.czbiohub.org/
UMAP v0.2.9	McInnes et al.^66^	https://cran.r-project.org/package=umap

**Other**		

Agilent 2100 Bioanalyzer	Agilent Technologies	FDA shared resource facility
BD Rhapsody Scanner	BD Biosciences	FDA shared resource facility
BD Rhapsody^TM^ Express single-cell analysis system	BD Biosciences	FDA shared resource facility
BD-LSRFortessa^TM^ X-20 Cell Analyzer	BD Biosciences	FDA shared resource facility
Illumina NovaSeq-6000	Illumina	Emory Integrated Genomics Core

### Experimental model and study participant details

#### Mouse

Age matched (5–7 weeks) female C57BL/6J were purchased from Charles River Laboratories (Germantown, MD). Mice were housed under specific pathogen-free conditions and maintained under standard environmental parameters for this species in an AAALAC-accredited facility at the U.S. Food and Drug Administration (FDA), Center for Biologics Evaluation and Research (CBER). All animal procedures were approved by the Institutional Animal Care and Use Committee (IACUC) at CBER (ASP 2010#03). Further, the animal protocol is in full accordance with ‘The guide for the care and use of animals’ as described in the US Public Health Service policy on Humane Care and Use of Laboratory Animals 2015 (chrome-extension://efaidnbmnnnibpcajpcglclefindmkaj/https://olaw.nih.gov/sites/default/files/PHSPolicyLabAnimals.pdf).

#### Parasites

Trypomastigotes of the *T. cruzi* Colombiana strain (DTU TcI) expressing nanoluciferase^10^ were harvested from culture supernatants of infected LLC-MK2 cells. Parasite numbers were determined using a Cellometer K2 Fluorescent Viability Cell Counter (Nexcelom Bioscience, MA).

### Method details

#### Experimental infection of mice

Mice were infected intraperitoneally with 10^4^ cell culture derived TcCOL-NLuc trypomastigotes.^10^ Peripheral blood parasitemia was assessed by light microscopy through quantification of parasites in an unstained 5 μL blood aliquot collected from the tail vein. The number of parasites was estimated as described.^67^ Mice were euthanized using a CO2 chamber at 110 days post infection (dpi).

#### *Ex-vivo* bioluminescence imaging

Infected mice were sacrificed at 110 days post-infection (dpi) by exsanguination under terminal anesthesia, followed by cardiac perfusion with 10 mL of PBS.^11^ Colons were dissected, transferred to Petri dishes, rinsed in PBS, and incubated in a furimazine solution (Promega) for 5 min. Images were acquired with exposure times ranging from 30 s to 2 min, depending on signal intensity. Colon tissues were then washed in PBS and dissociated into single-cell suspensions, as described later in discussion.

#### Isolation of colonic lamina propria cells

To prepare single-cell suspensions of lamina propria (LP) cells, colons from naive and infected mice were harvested immediately after euthanasia and rinsed with cold phosphate-buffered saline (PBS) supplemented with 2% fetal bovine serum (FBS) to remove fecal content. Peyer’s patches and surrounding fat were carefully excised. The tissue was then opened longitudinally to expose the lumen and cut into approximately 0.5 cm fragments. The resulting pieces were transferred to 50 mL conical tubes and subjected to a pre-digestion step in Hank’s Balanced Salt Solution (HBSS, without Ca^2+^ and Mg^2+^) containing 10 mM HEPES, 5 mM EDTA, 5% FBS, and 1 mM dithiothreitol (DTT). Samples were incubated at 37 °C for 20 min with continuous shaking at 200 rpm. Following incubation, the suspension was passed through a 70 μm cell strainer to remove epithelial cells and debris. The remaining material was transferred to gentleMACS C Tubes (Miltenyi Biotec) and subjected to enzymatic digestion for 30 minutes at 37 °C with shaking (200 rpm) in a buffer containing HBSS (with Ca^2+^ and Mg^2+^), 10 mM HEPES, 5% FBS, collagenase type I (2 mg/mL, Thermo Fisher Scientific), collagenase type II (2 mg/mL, Thermo Fisher Scientific), dispase (1 U/mL, Thermo Fisher Scientific), and DNase I (0.5 mg/mL, Sigma-Aldrich). For all flow cytometry experiments, enzymatic digestion was performed using Enzymes A, D, and R from the Lamina Propria Dissociation Kit (Miltenyi Biotec). Following digestion, the cell suspension was processed using the gentleMACS Dissociator (Miltenyi Biotec; program: *m_intestine_01*) and passed through a 70 μm strainer. Immune lamina propria cells were then isolated from the interface of a 40%/80% Percoll density gradient (Cytiva). Cell number and viability were assessed using ViaStain AOPI staining solution (Revvity, Inc.) and quantified with a Cellometer K2 Fluorescent Viability Cell Counter (Revvity, Inc.).

#### T cell purification

Lamina propria single-cell suspensions were prepared as described above. CD8^+^ T cells were isolated using Miltenyi CD8a (Ly-2) MicroBeads according to manufacturer’s instructions. Briefly, 10^7^ LP cells were resuspended in MACS buffer (PBS, 0.5% BSA, 2 mM EDTA) and incubated with 20 μL MicroBeads at 4°C for 15 min. Cells were washed and applied to an LS column. After three washes, CD8^+^ T cells were then eluted and immediately processed for flow cytometry to ensure viability.

#### Preparation of libraries and single cell RNA-seq

Single-cell suspensions of LP cells, isolated from three infected and three naive mice, were pooled into two experimental groups. Cell capture and cDNA library preparation were performed using the BD Rhapsody Express Single-Cell Analysis System^20^ (BD Biosciences) in accordance with the manufacturer’s instructions. Prior to viability and concentration assessment using the BD Rhapsody Scanner, cells were stained with Calcein AM (Thermo Fisher Scientific) and Draq7 (BD Biosciences). Stained cells were then loaded onto a primed BD Rhapsody Cartridge and processed on the BD Rhapsody Express system. Magnetic barcoded beads were subsequently added to the cartridge and incubated to capture single cells within individual microwells. Following *in situ* cell lysis, beads carrying cell-associated mRNA were collected for reverse transcription using the BD Rhapsody cDNA Synthesis Kit and oligo(dT) primers. Whole transcriptome libraries were generated with the BD Rhapsody Whole Transcriptome Analysis (WTA) Amplification Kit, producing full-length cDNA tagged with cell-specific barcodes and unique molecular identifiers (UMIs). The resulting cDNA was amplified by PCR, purified using AMPure XP beads (Beckman Coulter), and quantified with a Qubit 4 Fluorometer (Thermo Fisher Scientific) using the Qubit dsDNA High Sensitivity (HS) Assay Kit (Thermo Fisher Scientific). Library quality was assessed on an Agilent 2100 Bioanalyzer (Agilent Technologies). Final WTA cDNA libraries were pooled and sequenced on the NovaSeq 6000 system (Illumina) at the Emory Integrated Genomics Core.

#### Curation of custom reference for single cell RNA-seq libraries

For single cell analysis, genomic sequences and GTF files for *Mus musculus* (assembly GRCm39 with accession number GCF_000001635.27) were merged with those of *Trypanosoma cruzi* (assembly ASM371945v1.61 with accession number GCA_003719455) to generate a custom reference genome. *T. cruzi* annotations were appended to the mouse reference and used for alignment with the BD Rhapsody WTA scRNA-seq libraries to generate BAM files and gene-cell count matrices.

#### Single cell RNA-seq data analysis

Count matrices generated by BD Rhapsody were analyzed in R (v4.3.3)^62^ using Seurat (v4.4).^63^ Cells with fewer than 500 detected genes or >10% mitochondrial gene content were excluded. Doublets were identified using scDblFinder^64^ based on unique molecular identifiers (UMIs) and removed. To identify infected cells, we calculated the proportion of sequencing reads mapping to *T. cruzi* transcripts for each single cell. Cells with >1% parasite reads were classified as infected. Cells below this threshold were not reliably distinguishable from uninfected cells. Samples were normalized using SCTransform and integrated using Seurat’s SCTransform-based integration workflow,^63^ retaining 3,000 variable features. Dimensionality reduction was performed by principal component analysis (PCA), and the first 30 PCs were used to construct a K-nearest neighbors graph followed by Louvain clustering with a resolution of 0.5. Cells were visualized using Uniform Manifold Approximation and Projection (UMAP).^66^ Cluster annotation to biological cell types was performed manually based on canonical marker gene expression using CellMarker,^58^ CellMarker 2.0,^59^ panglaoDB,^61^ Tabula Muris^65^ reference databases. Marker genes for each population were identified using the Wilcoxon rank-sum test (Seurat v4.4), comparing each cluster against all other cells. Only genes expressed in at least 10% of cells in either the target cluster or the comparison group were considered. *p* values were adjusted using the Benjamini-Hochberg method.

#### Cell communication network (CCN) analysis

Cell–cell communication analysis was performed using CellChat (v1)^21^ to infer and quantify signaling networks from single-cell RNA-sequencing. The secreted signaling database was used for all analyses. CellChat utilizes a curated ligand–receptor interaction database and a mass-action–based model to compute communication probabilities based on gene expression levels. Overexpressed ligands and receptors were first identified across cell populations. Communication probabilities were then computed for each ligand–receptor pair. Pairs not detected in each sample but present in others were assigned a communication probability of zero prior to downstream analysis. Individual ligand–receptor interactions were aggregated into pathway-level communication networks, and interaction strengths were averaged across samples.

#### Differential gene expression and pathway analysis

Differential gene expression (DGE) analysis was performed for CD8^+^ cytotoxic T cell and macrophage clusters using the DESeq2^60^ R package. Read counts were normalized using the median-of-ratios method. Genes with a Benjamini–Hochberg-adjusted *p*-value <0.05 and an absolute fold change ≥1.5 were considered significantly differentially expressed. Log_2_ fold-change values and adjusted *p*-values were uploaded into Ingenuity Pathway Analysis (IPA, Qiagen) to identify enriched canonical pathways. Pathway activation or inhibition was inferred using z-scores (|z| ≥ 2), and statistical significance was assessed using a right-tailed Fisher’s exact test (*p* < 0.05).

#### Flow cytometry

Flow cytometry analysis was performed to identify cell populations based on the expression of cell surface markers using specific panels of fluorescently conjugated antibodies. Cells were first incubated with Ghost Dye Violet 510 fixable viability dye (Cell Signaling Technology) or Fixable Viability Stain 575V (BD Biosciences) to exclude dead cells, followed by a blocking step with Mouse BD Fc Block (BD Biosciences) to minimize nonspecific binding of antibodies to Fc receptors. The following antibody panels were used: a) to identify CD3^+^ T cells (Figure 2C), anti-CD3 BV421 from BD Biosciences; b) to identify macrophages (Figure 2E), anti-CD45 FITC and anti-F4/80 BV421 from BioLegend; c) to identify CD3^+^ CD8^+^ T cells (Figure 3C), anti-CD3 BV421 from BD Biosciences and anti-CD8 APC from Miltenyi Biotec; d) To identify T follicular helper cells (Figure 3E), anti-CD3 PE-CF594, anti-CXCR4 PE and anti-PD-1 from BD Biosciences, e) To identify CD3^+^ CD4^+^ CCR5^+^ T cells (Figure 5C), anti-CD3 BV421 from BD Biosciences, anti-CD4 APC from Miltenyi Biotec and anti-CCR5 FITC from BioLegend. Antibodies were diluted to ≤0.25–0.5 μg per 1 × 10^6^ cells. After staining, cells were fixed with fixation buffer (BD Biosciences) according to the manufacturer’s instructions, washed, and resuspended in PBS. Data acquisition was conducted on a BD LSRFortessa X-20 Cell Analyzer (BD Biosciences), and compensation was performed with appropriate single-color controls. Flow cytometry data were analyzed using FlowJo v10. 10 software (BD Biosciences), and gating strategies were based on fluorescence-minus-one (FMO) controls to accurately define positive populations.

#### Quantitative RT-PCR

Total RNA was purified from LP cells using the PureLink RNA Mini Kit (Thermo Fisher Scientific). Following purification, RNA was reverse transcribed into cDNA using the iScript gDNA Clear cDNA Synthesis Kit (Bio-Rad Laboratories) with 0.5–1 μg of RNA template. Quantitative PCR was performed using the SsoAdvanced Universal SYBR Green Supermix (Bio-Rad Laboratories) on a CFX96 Touch Real-Time PCR Detection System (Bio-Rad Laboratories). The cycling protocol consisted of an initial activation step at 95°C for 2 minutes, followed by 40 cycles of 5 s at 95°C and 30 s at 60°C. A melt curve analysis was then performed, with 5-s from 65°C to 95°C in 0.5°C increments. Data were analyzed using CFX Manager Software. Gene expression was quantified using the ΔCt method, with normalization to GAPDH (mouse) as the reference gene.^68^

### Quantification and statistical analysis

Statistical differences between group means were evaluated using an unpaired two-tailed Student’s *t* test, performed with GraphPad Prism version 10.1.2. The number of replicates for each experiment is indicated in the corresponding figure legends. All bar graphs display the mean ± standard deviation (SD). A *p*-value of <0.05 was considered statistically significant.

## Supplemental information

Document S1. Figures S1–S5

Table S1. Predicted ligand–receptor interactions in the colon of naive mice identified by CellChat, related to Figure 4 provided as Excel file Table S1

Table S2. Predicted ligand–receptor interactions in the colon of chronically *T. cruzi* - infected mice identified by CellChat, related to Figure 4 provided as Excel file Table S2

Table S3. Differential signaling pathway communication probabilities between naive and chronically *T. cruzi* - infected mice inferred by CellChat, related to Figure 4, provided as Excel file Table S3

Table S4. Top CellChat-inferred signaling pathway communication probabilities between naive and chronically *T. cruzi* - infected mice, related to Figure 4A, provided as Excel file Table S4

Table S5. Differentially expressed genes in colonic CD8^+^ cytotoxic T cells from infected and naive mice, related to Figure 6A provided as Excel file Table S5

Table S6. Differentially expressed genes in colonic macrophages from infected and naive mice, related to Figure 6D provided as Excel file Table S6
