# Supplementary material for: CCL signaling drives T Cell–Macrophage crosstalk in the mouse colon during chronic Trypanosoma cruzi infection
Source: iScience. 2026 Jul 2;29(7):116611. doi: 10.1016/j.isci.2026.116611 (PMC13355016; doi:10.1016/j.isci.2026.116611)
Supplement: Document S1. Figures S1–S5 [file mmc1.pdf]

iScience, Volume *29*

Supplemental information

CCL signaling drives T Cell–Macrophage crosstalk in the mouse colon during chronic *Trypanosoma cruzi* infection

Erica Silberstein, Spyros Karaiskos, Nikki Tirrell, Charles C. Chung, Supriya Kumar, Jung-Sun Cho, and Alain Debrabant

Figure S1

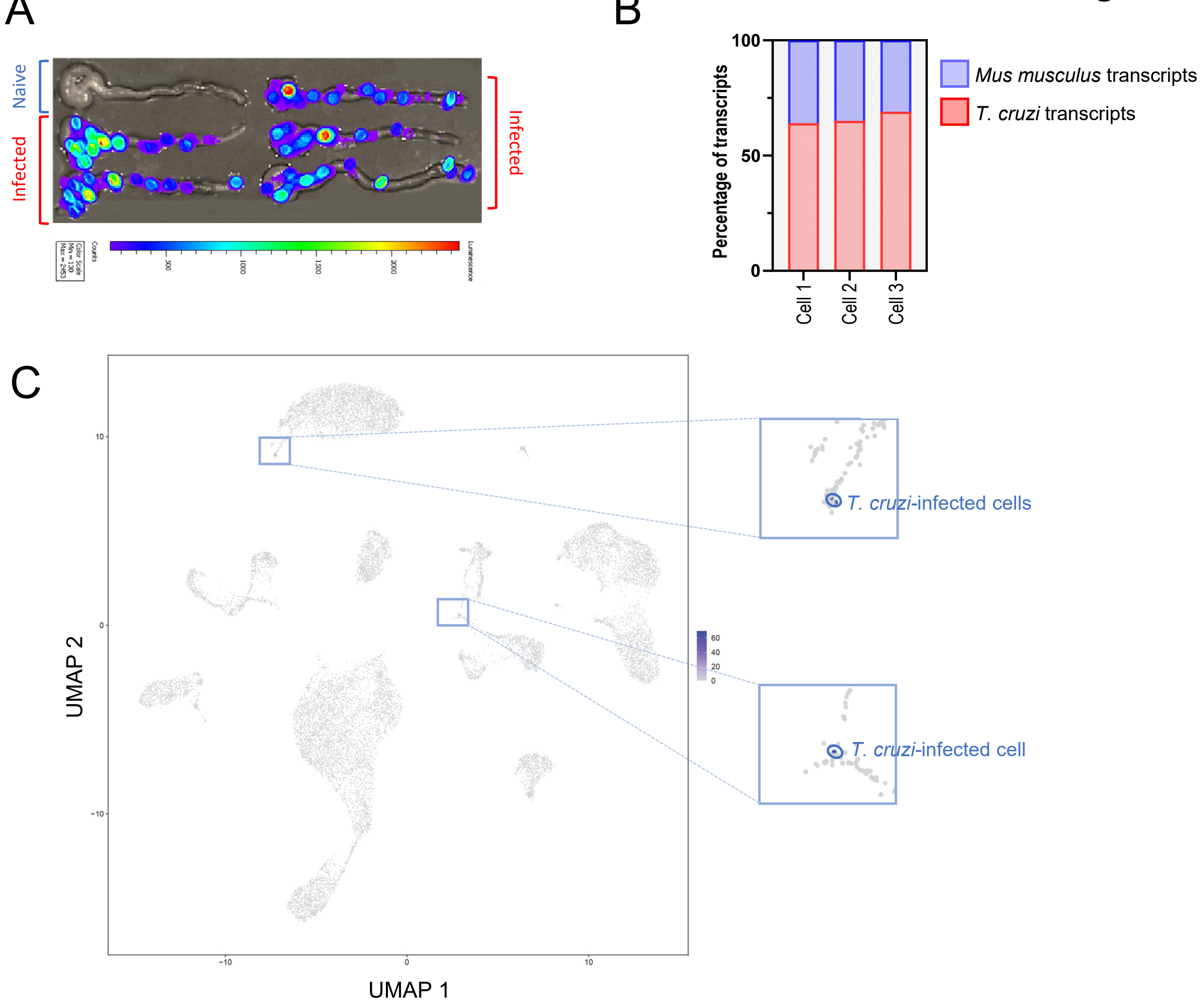

Figure S1. Bioluminescence imaging and single-cell transcriptomic analysis of *T. cruzi*-infected cells in mouse colon tissue. (A) Naïve and TcCOL-NLuc-infected selected mice were sacrificed 110 dpi. Colon tissues were excised and transferred to a 6-well plate. After rinsing with PBS, samples were soaked in a furimazine solution for 5 minutes. Images were acquired using an exposure time from 30 seconds to 2 minutes. Log 10 heat-map scales represent bioluminescence intensity (blue: low; red: high). (B) Bar graph showing the proportion of transcripts derived from *T. cruzi* infected cells. (C) Uniform Manifold Approximation and Projection (UMAP) plot displaying ~16,000 cells isolated from mouse colons, with *T. cruzi*-infected cells highlighted in blue.

Figure S2

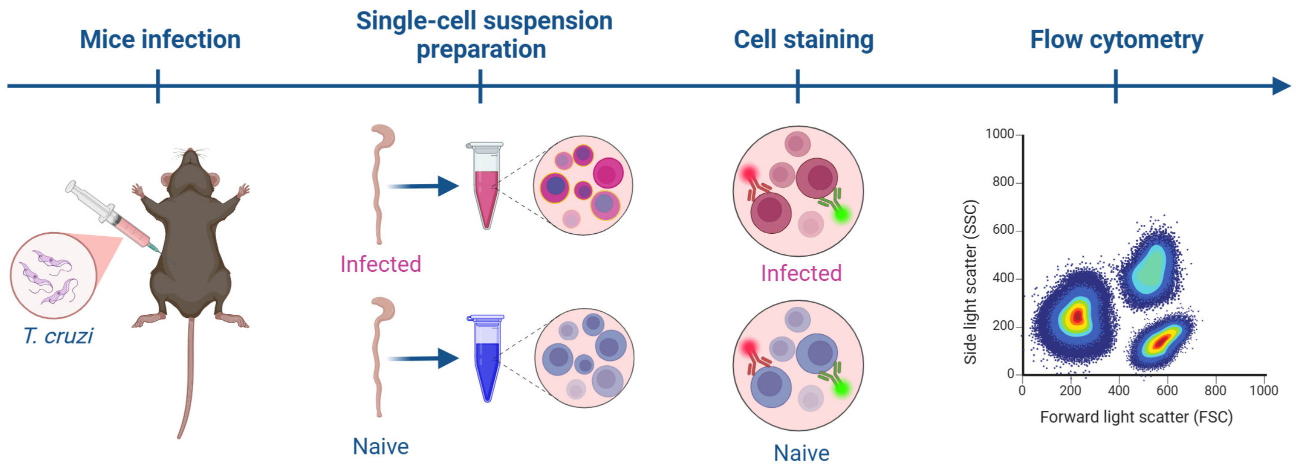

Figure S2. Experimental Workflow for Flow Cytometry-Based Analysis. C57BL/6 mice were infected intraperitoneally (i.p.) with 10^4 *Trypanosoma cruzi* Colombiana parasites expressing nanoluciferase. Lamina propria cells were isolated from the colon of both infected and naïve mice and stained with cell-specific antibody panels. Flow cytometry acquisition and data analysis were subsequently performed to quantify immune cell populations.

Figure S3

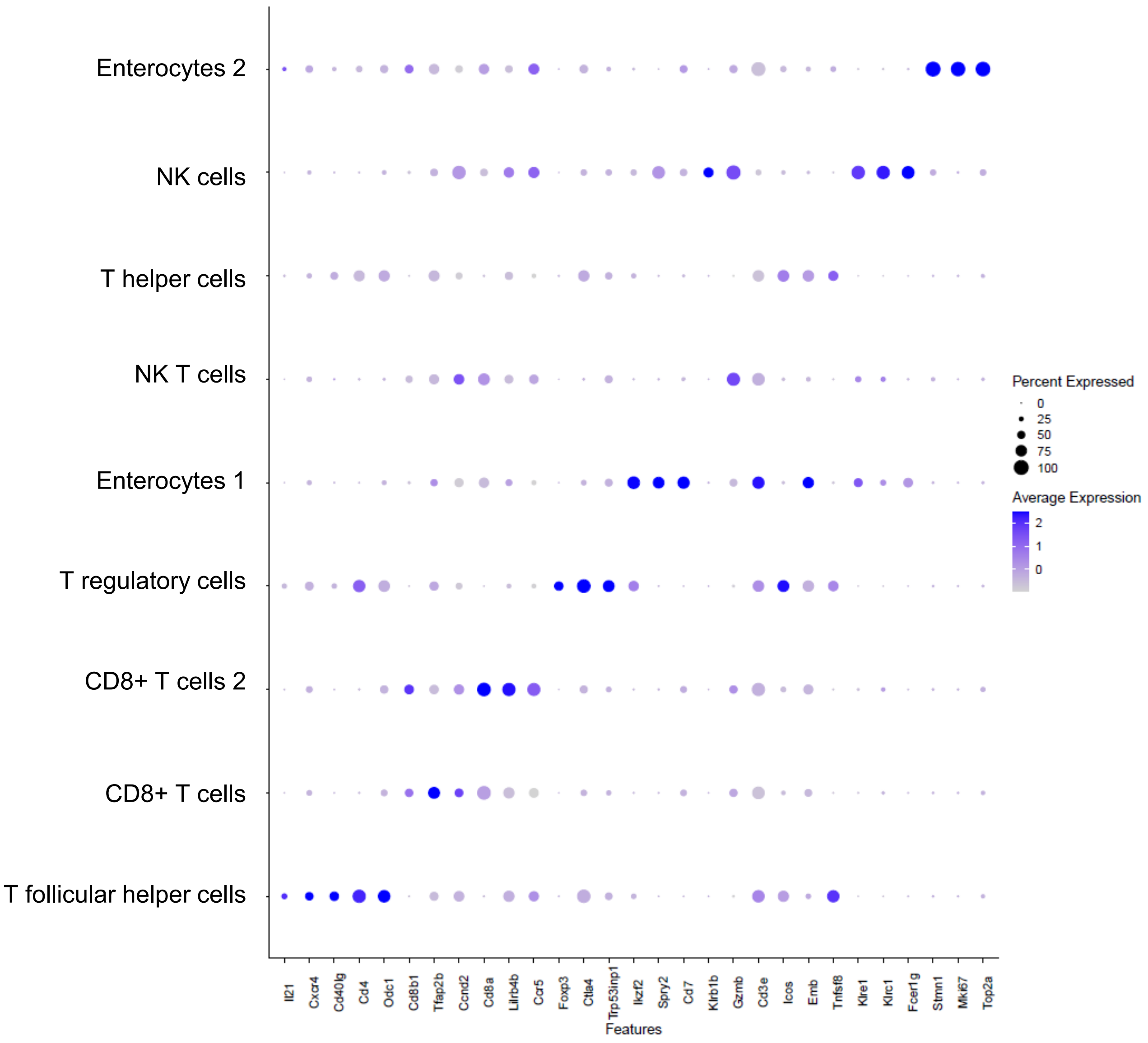

Figure S3. Identification of T cell subsets within the T and NK cells cluster. Expression levels of the top cluster-specific genes used to annotate T cell populations. Dot size represents the percentage of cells expressing each gene, while dot color indicates the expression level.

Figure S4

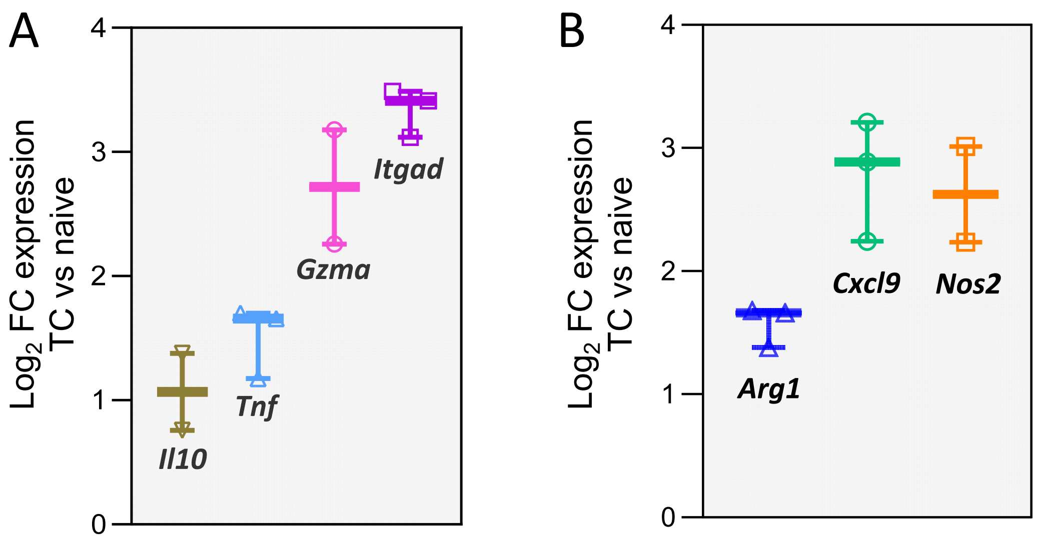

Figure S4. mRNA quantification of selected upregulated genes in *T. cruzi*-chronically infected mice. The plots display the log2 fold changes (FC) in gene expression, calculated using the ΔΔCt method and normalized to GAPDH expression. The horizontal line indicates the median value for each group, with individual data points plotted. The vertical lines represent the error bars. The assay was performed in two (*Il10*, *Gzma* and *Nos2*) or 3 (*Tnf*, *Itgad, Arg1* and *Cxcl9*) independent experiments pooling cells from 3 naïve and 3 infected mice. (A) $CD8^+$ T cytotoxic cells genes. (B) Macrophages genes.

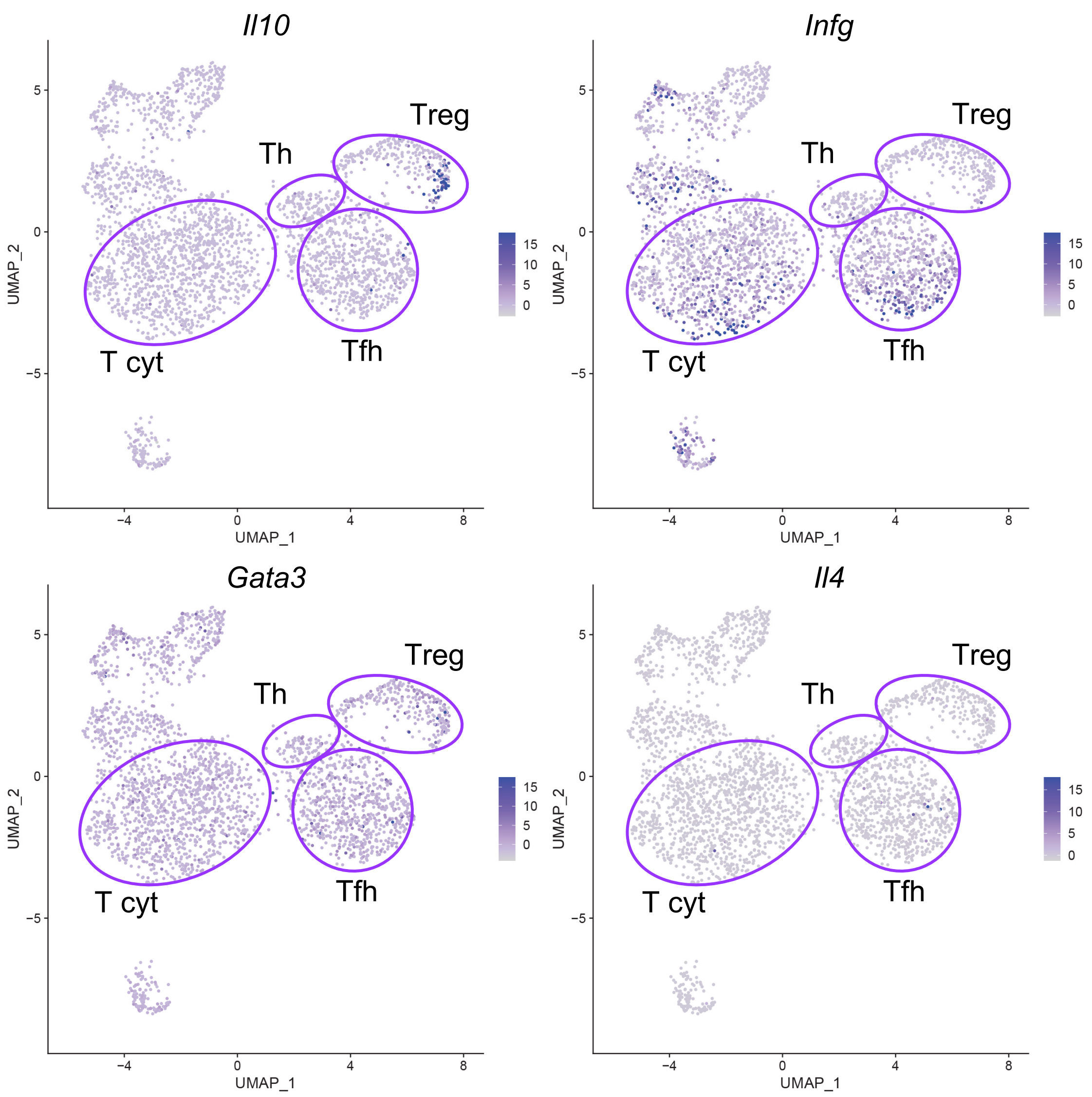

Figure S5. UMAP plot showing *Il-10*, *Ifn-g*, *Gata 3* and *Il-4* expression across colonic T cell subclusters. Color scale indicates normalized gene expression levels. T cyt: Cytotoxic T cells; Tfh: T follicular helper cells; Th: T helper cells; Treg: T regulatory cells.
